# Editorial: Incorporation of texture analysis in diagnosing and characterizing cancer

**DOI:** 10.3389/fonc.2023.1224644

**Published:** 2023-06-06

**Authors:** Junbang Feng, Chuanming Li

**Affiliations:** ^1^ Chongqing University Central Hospital, Chongqing, China; ^2^ Chongqing Emergency Medical Center, Chongqing, China

**Keywords:** texture analysis, radiomics, CT, MRI, cancer

Cancer is a group of diseases which are aggressive, invasive, and sometimes metastatic. It can affect people of all ages, even fetuses, and the risk increases with age. According to the statistics of the American Cancer Society, it is estimated that there will be more than 600000 cancer deaths in the United States in 2023. Currently, *in vivo* diagnosis and evaluation of cancer mainly rely on methods of medical imaging such as MRI and CT. However, many small and hidden information in MRI and CT can not be detected by naked eye observation. Radiomics is a new technology that can be used to automatically mine large amounts of high-throughput data features from medical images. Although many studies have reported the use of radiomics and texture analysis in the diagnosis of cancer in recent years, further exploration of their potential in early cancer discrimination and prognosis is still worth exploring.

In this Research Topic, the application of radiomics and texture analysis in the diagnosis, differentiation, and prognostic evaluation of various cancers was focused. Texture analysis could reflect the physiological and pathological changes of cancer by extracting a large number of potential image features. The signal intensity features could reflect the composition of a tissue, while texture features could reflect the consistency of internal components within the tissue. Sun et al. investigated the potential of CT imaging features and texture analysis to distinguish bronchiolar adenoma from adenocarcinoma *in situ* and minimally invasive adenocarcinoma. They found that the multiple logistic regression model based on six key texture features extracted from non-enhanced CT could accurately identify them with the AUC of 0.977 and 0.976, respectively. The Six key texture features were GLCMEntropy, LongRunLowGreyLevelEmphasis, GLCMEnergy, ShortRunEmphasis VoxelValueSum, and Quantile. Q. Some pulmonary nodules may gradually/grow and develop into lung cancer, while others may remain stable for many years. Accurately predicting the growth of pulmonary nodules in advance is of great clinical significance for early treatment (Shen et al.). The SVM, RF, MLP and AdaBoost models developed by Yang et al. based on age and radiomic features demonstrated high accuracy in predicting the growth of pulmonary nodules in the future next year, with AUCs of 0.81, 0.77, 0.81 and 0.71 respectively in the validation group. The rearrangement state of anaplastic lymphoma kinase (ALK) plays a key role in targeted therapy of non-small cell lung cancer. However, how to accurately detect them is a great challenge. Hao et al. proved that ALK rearrangement status could be accurately predicted using a machine learning-based classification model based on CT textural features and clinical data. This study provided a non-invasive solution for accurately identifying ALK gene status, which was an efficient and rapid new method for clinical genetic diagnosis. Pancreatic ductal adenocarcinoma (PDAC) accounts for more than 90% of pancreatic cancer and is the seventh leading cause of cancer-related death worldwide. Early identification of lymph node metastasis around PDAC is very important for surgical treatment. Li et al. successfully constructed a logistic regression model to detect lymph node metastasis using radiomics features of enhanced CT, and the AUC of the training group and the validation group were 0.937 and 0.851, respectively. This model may help clinicians accurately assess the risk of lymph node metastasis before surgery. Liver cancer is the sixth most common cancer in the world and poses a serious threat to human health. Artificial intelligence and radiomics has undergone rapid development and has a wide application in the diagnosis and treatment of liver diseases. Xiong et al. compiled a relative comprehensive and quantitative report on the research of liver disease using artificial intelligence by employing bibliometrics. They summarized the current research progress, hotspots, and emerging trends of cancer artificial intelligence. The microvascular invasion (MVI) refers to the tumor invasion in small intrahepatic vessels, covering portal veins, hepatic vessels, and lymphatic vessels. Zhang et al. developed a nomogram to predict preoperative MVI of HCC with an AUC of 0.884 by combining clinicoradiological factors and radiomics features. This was crucial for accurately identifying the malignancy of HCC and developing appropriate treatment plans. Endometrial cancer (EC) is one of the three most common malignant tumors in the female reproductive system. Its incidence rate and mortality are on the rise and spread to young people. Ki-67 and p53 are closely related to the proliferation and apoptosis of tumor cells. Jiang et al. established a logistic regression model based on the textural features and apparent diffusion coefficient values of multimodal MRI to predict the expression levels of Ki-67 and p53 before surgery. In the verification group, the AUCs were 0.938 and 0.922, which had high auxiliary diagnostic value for clinical application. Their method could non-invasive evaluate the levels of Ki 67 and p53 in EC, and had important potential in evaluating the malignancy of EC and guiding appropriate treatment plans. Prostate cancer (PCa) occurs in middle-aged men aged 45-60 years and is the most common malignant tumor of the male reproductive system. High-risk prostate cancer (PCa) is often treated by prostate-only radiotherapy (PORT) owing to its favourable toxicity profile compared to whole-pelvic radiotherapy. Unfortunately, more than 50% patients still developed disease progression following PORT. Ching et al. developed a Ridge regression model based on radiomics and clinical characteristics of preoperative planned computed tomography, which could predict the progression of high-risk PCa patients at 5 years after prostate-only radiotherapy with an AUC of 0.797. This study might help clinicians for the personalized treatment of high-risk prostate cancer patients in the future.

Through this Research Topic, the important role of texture analysis in cancer imaging was clearly presented, which provided quantitative and objective support for various cancer detection and treatment decision. Although there are still many difficulties in achieving a complete cure for cancer, new treatment methods such as targeted drugs have brought many new hopes. Radiomics and texture analysis can greatly assist in the diagnosis, gene prediction, and prognosis evaluation of tumors, and have the hope of being used as a decision-making tool in future clinical applications.

## Author contributions

CL and JF: conceptualization; data curation; manuscript drafting; manuscript editing. All authors approved the submitted version.

